# Supplementary material for: Mesmerize is a dynamically adaptable user-friendly analysis platform for 2D and 3D calcium imaging data
Source: Nat Commun. 2021 Nov 12;12:6569. doi: 10.1038/s41467-021-26550-y (PMC8589933; doi:10.1038/s41467-021-26550-y)
Supplement: Supplementary file 3 — Reporting Summary [file 41467_2021_26550_MOESM3_ESM.pdf]

nature portfolio

Corresponding author(s): Kushal Kolar, Marios Chatzigeorgiou

Last updated by author(s): Sep 29, 2021

Reporting Summary

Nature Portfolio wishes to improve the reproducibility of the work that we publish. This form provides structure for consistency and transparency in reporting. For further information on Nature Portfolio policies, see our Editorial Policies and the Editorial Policy Checklist.

Statistics

For all statistical analyses, confirm that the following items are present in the figure legend, table legend, main text, or Methods section.

n/a	Confirmed	
☐	☒	The exact sample size (*n*) for each experimental group/condition, given as a discrete number and unit of measurement
☐	☒	A statement on whether measurements were taken from distinct samples or whether the same sample was measured repeatedly
☒	☐	The statistical test(s) used AND whether they are one- or two-sided<br>*Only common tests should be described solely by name; describe more complex techniques in the Methods section.*
☒	☐	A description of all covariates tested
☒	☐	A description of any assumptions or corrections, such as tests of normality and adjustment for multiple comparisons
☐	☒	A full description of the statistical parameters including central tendency (e.g. means) or other basic estimates (e.g. regression coefficient) AND variation (e.g. standard deviation) or associated estimates of uncertainty (e.g. confidence intervals)
☒	☐	For null hypothesis testing, the test statistic (e.g. *F*, *t*, *r*) with confidence intervals, effect sizes, degrees of freedom and *P* value noted<br>*Give P values as exact values whenever suitable.*
☒	☐	For Bayesian analysis, information on the choice of priors and Markov chain Monte Carlo settings
☒	☐	For hierarchical and complex designs, identification of the appropriate level for tests and full reporting of outcomes
☒	☐	Estimates of effect sizes (e.g. Cohen's *d*, Pearson's *r*), indicating how they were calculated

Our web collection on statistics for biologists contains articles on many of the points above.

Software and code

Policy information about availability of computer code

Data collection	All data were collected using a custom analysis program which openly available on GitHub and has been deposited in Zenodo.<br>Kolar, Kushal, & Chatzigeorgiou, Marios. (2019, August 18). Simple GUI for acquiring images from a Hamamatsu Orca Flash 4.0 CMOS camera (Version 0.1.0). Zenodo. http://doi.org/10.5281/zenodo.3370464
Data analysis	GitHub repo for Mesmerize: https://github.com/kushalkolar/MESmerize<br>Zenodo DOI for Mesmerize GitHub repo archive: https://doi.org/10.5281/zenodo.5539440<br>Notebooks that produce some of the figures are available on GitHub:<br>https://github.com/kushalkolar/mesmerize_manuscript_notebooks<br>Many of these notebooks can be run on MyBinder:<br>https://mybinder.org/v2/gh/kushalkolar/mesmerize_manuscript_notebooks/master<br>Mesmerize can be installed through pip on all platforms:<br>https://pypi.org/project/mesmerize/<br>We provide a ready to use VM with Mesmerize and all features pre-installed. You can run this VM on Windows, Mac OSX, or Linux. Please visit:<br>http://docs.mesmerizelab.org/en/master/user_guides/installation.html#all-platforms<br>Thorough Mesmerize documentation can be found here:<br>http://docs.mesmerizelab.org/<br>Software packages mentioned in the manuscript for comparisons:<br>OMERO v5.6<br>Biaflows, versioning is unclear<br>Cytomine v3<br>openBIS v20.10

KNIME v4.3
EZCalcium v2.1.1
SIMA v1.3
S.A. Romano package, does't use versioning
DataJoint v0.13
Python packages mentioned in the manuscript that are used by Mesmerize:
caiman v1.8.4
tslearn v0.4.1
pyqtgraph v0.10.0
numpy v1.19.0
pandas v0.25.3
matplotlib v3.2.1
nuset-lib v0.1.1
PyQt5 v5.9.2
scikit-learn v0.23.1
scipy v1.2.1
Software packages not used but have interoperable data with Mesmerize:
suite2p v0.8

For manuscripts utilizing custom algorithms or software that are central to the research but not yet described in published literature, software must be made available to editors and reviewers. We strongly encourage code deposition in a community repository (e.g. GitHub). See the Nature Portfolio guidelines for submitting code & software for further information.

Data

Policy information about availability of data

All manuscripts must include a data availability statement. This statement should provide the following information, where applicable:

- Accession codes, unique identifiers, or web links for publicly available datasets
- A description of any restrictions on data availability
- For clinical datasets or third party data, please ensure that the statement adheres to our policy

The datasets are available on figshare and contain the raw data, analysis procedures, and plots in a FAIR-functionally linked system as described in the paper.
C. intestinalis: https://doi.org/10.6084/m9.figshare.10289162
Zebrafish dataset as a Mesmerize dataset: https://doi.org/10.6084/m9.figshare.14748915
PVC-7 as a Mesmerize dataset: https://doi.org/10.6084/m9.figshare.10293041

Field-specific reporting

Please select the one below that is the best fit for your research. If you are not sure, read the appropriate sections before making your selection.

[x] Life sciences [ ] Behavioural & social sciences [ ] Ecological, evolutionary & environmental sciences

For a reference copy of the document with all sections, see nature.com/documents/nr-reporting-summary-flat.pdf

Life sciences study design

All studies must disclose on these points even when the disclosure is negative.

Sample size	We achieved a high agglomerative coefficient from the clustering of our samples and drew our biological conclusions from the number of clusters indicated by Silhouette and Davies Bouldin scores. Since this was an initial exploratory study into the calcium dynamics of neuronal & non-neuronal cells of C. intestinalis without any treatment groups to alter the calcium dynamics further sample-size calculations were not necessary.
Data exclusions	CNMFE extracted signals that represented movement in the FOV or noise were excluded. Signals from heavily out of focus regions or cells were also excluded.
Replication	All attempts at replication were successful. All replicates were included in our analysis. The animals used in our analysis were the outcome of at least two independent electroporations per promoter construct. Number of animals used per promoter are shown in Supplementary Table 1.
Randomization	Not relevant, animals electroporated with a particular promoter>GCaMP6s construct belong to that group.
Blinding	Investigators were not blinded to experimental groups (promoter>GCaMP6s) during data collection. Blinding would have been difficult because the reporters have easily recognizable expression patterns. However, since we were not using manual ROI selection but instead used CNMF, the resulting data came from a process that is not aware of the group (promoter>GCaMP6s).

Reporting for specific materials, systems and methods

We require information from authors about some types of materials, experimental systems and methods used in many studies. Here, indicate whether each material, system or method listed is relevant to your study. If you are not sure if a list item applies to your research, read the appropriate section before selecting a response.

Materials & experimental systems

n/a	Involved in the study
☒	☐ Antibodies
☒	☐ Eukaryotic cell lines
☒	☐ Palaeontology and archaeology
☐	☒ Animals and other organisms
☒	☐ Human research participants
☒	☐ Clinical data
☒	☐ Dual use research of concern

Methods

n/a	Involved in the study
☒	☐ ChIP-seq
☒	☐ Flow cytometry
☒	☐ MRI-based neuroimaging

Animals and other organisms

Policy information about studies involving animals; ARRIVE guidelines recommended for reporting animal research

Laboratory animals	No laboratory animals were used. The experiments relating to the pvc-7 mouse dataset that we downloaded from CRCNS were performed by the Allen Institute for Brain Sciences. Detailed information on the experiment including animal handling can be found here: http://crcns.org/files/data/pvc-7/allen_inst_ophys_summary.pdf . The experiments relating to the zebrafish dataset that were provided by Martin Haesemeyer are described in the following paper: Haesemeyer M, Robson DN, Li JM, Schier AF, Engert F. A Brain-wide Circuit Model of Heat-Evoked Swimming Behavior in Larval Zebrafish. Neuron. 2018 May 16;98(4):817-831.e6. doi: 10.1016/j.neuron.2018.04.013. Epub2018 May 3. PMID: 29731253; PMCID: PMC5985529. All experiments followed the guidelines of the National Institutes of Health and were approved by the Standing Committee on the Use of Animals in Research of Harvard University.
Wild animals	Ciona intestinalis gravid hemaphrodite adults were collected from Bildøy Marina, Bergen, Norway. The adults were between 4 and 8 months old. The adults were manually removed from their sites of settlement (ropes, pier-piles, boat jetis) placed in 10L containers with sea water and transported by car to our facility. To generate larvae, adult animals were dissected in order to collect sperm and eggs. Sacrificed adults were taken for incineration according to University of Bergen regulations.
Field-collected samples	Ciona intestinalis animals were kept in a purpose built Genetically Modified Organism (GMO) certified facility. The animals were placed in 50L tanks with circulating sea water from the ocean, at 10 degrees Celcius (in order to maintain similar temperature conditions to those in the ocean), with constant illumination to prevent spawning. The animals were fed daily a diet of algae and spirulina cyanobacteria. Transgenic Ciona intestinalis used in our experiments were disposed in our GMO approved facility in accordance with University of Bergen regulations.
Ethics oversight	No ethical approval was required for the use of Ciona intestinalis since this is an invertebrate animal and it is not covered by the existing animal acts.

Note that full information on the approval of the study protocol must also be provided in the manuscript.
